# Nur77 Decreases Atherosclerosis Progression in apoE^−/−^ Mice Fed a High-Fat/High-Cholesterol Diet

**DOI:** 10.1371/journal.pone.0087313

**Published:** 2014-01-31

**Authors:** Yan-Wei Hu, Peng Zhang, Jun-Yao Yang, Jin-Lan Huang, Xin Ma, Shu-Fen Li, Jia-Yi Zhao, Ya-Rong Hu, Yan-Chao Wang, Ji-Juan Gao, Yan-Hua Sha, Lei Zheng, Qian Wang

**Affiliations:** 1 Laboratory Medicine Center, Nanfang Hospital, Southern Medical University, Guangzhou, Guangdong, China; 2 Department of Anesthesiology, Nanfang Hospital, Southern Medical University, Guangzhou, Guangdong, China; Wageningen University, Netherlands

## Abstract

**Rationale:**

It is clear that lipid disorder and inflammation are associated with cardiovascular diseases and underlying atherosclerosis. Nur77 has been shown to be involved in inflammatory response and lipid metabolism.

**Objective:**

Here, we explored the role of Nur77 in atherosclerotic plaque progression in apoE^−/−^ mice fed a high-fat/high cholesterol diet.

**Methods and Results:**

The Nur77 gene, a nuclear hormone receptor, was highly induced by treatment with Cytosporone B (Csn-B, specific Nur77 agonist), recombinant plasmid over-expressing Nur77 (pcDNA-Nur77), while inhibited by treatment with siRNAs against Nur77 (si-Nur77) in THP-1 macrophage-derived foam cells, HepG2 cells and Caco-2 cells, respectively. In addition, the expression of Nur77 was highly induced by Nur77 agonist Csn-B, lentivirus encoding Nur77 (LV-Nur77), while silenced by lentivirus encoding siRNA against Nur77 (si-Nur77) in apoE^−/−^ mice fed a high-fat/high cholesterol diet, respectively. We found that increased expression of Nur77 reduced macrophage-derived foam cells formation and hepatic lipid deposition, downregulated gene levels of inflammatory molecules, adhesion molecules and intestinal lipid absorption, and decreases atherosclerotic plaque formation.

**Conclusion:**

These observations provide direct evidence that Nur77 is an important nuclear hormone receptor in regulation of atherosclerotic plaque formation and thus represents a promising target for the treatment of atherosclerosis.

## Introduction

Coronary atherosclerosis represents the leading cause of morbidity and mortality of men and women throughout the western world. Hypercholesterolemia is a well-established risk factor for the incidence of atherosclerosis and its pathological complications [1]. Evidence from clinical trials indicates that reducing plasma cholesterol by dietary and/or pharmacological means leads to reductions in the incidence of death from cardiovascular disease [2], [3]. Appealing therapeutic targets for reducing hypercholesterolemia and atherosclerosis include members of the nuclear hormone receptor superfamily, such as the peroxisome proliferator–activated receptor and the liver X receptor subfamilies, among others. These ligand-activated transcription factors regulate various metabolic processes by controlling the expression of specific gene cassettes [4].

In addition to these well-characterized ligand-activated transcription factors, the nuclear receptor (NR) superfamily comprises many orphan receptors, whose ligands and physiological functions remain unknown. Among this group of orphan receptors is the NR4A subfamily, including Nur77 (NR4A1), Nurr1 (NR4A2), and Nor-1 (NR4A3). In contrast to other members of the superfamily, NR4A nuclear receptors are ‘immediate early genes’, and are transiently and rapidly induced by a pleiotropy of environmental cues [5]. Nur77 is a member of the NR4A subfamily and consists, like other nuclear receptors, of an N-terminal activating function-1 (AF-1) domain, a central two zinc-finger DNA-binding domain (DBD), and a C-terminal ligand binding domain (LBD) [6]. Early functional studies have pointed to a critical role of Nur77 in regulating differentiation, proliferation, and apoptosis. More recent research has characterized Nur77 as key transcriptional regulators of glucose and lipid homeostasis, adipogenesis, inflammation, and vascular remodeling. Initial experiments by Maxwell et al. demonstrated that Nur77 promotes lipolysis in muscle [7]. Subsequently, Pols et al. revealed that Nur77 modulates plasma lipoprotein profiles and hepatic lipid metabolism in mice [8]. Consistent with these data, Chao et al. noted hepatic steatosis and increased transcription factors sterol regulatory element-binding binding protein 1c (SREBP1c) expression in Nur77-deficient mice fed a high-fat diet [9].

The present study evaluated the effect of Nur77 on cholesterol metabolism in THP-1 macrophage-derived foam cells, and on plasma lipoprotein profiles, circulating cytokine levels, hepatic lipid deposition and the development of aortic atherosclerosis in apoE^−/−^ mice. Our findings demonstrated that Nur77 reduced lipid loading, lipid content and enhanced cholesterol efflux in THP-1 macrophage-derived foam cells. Furthermore, we found that Nur77 down-regulated levels of inflammatory molecules, adhesion molecules and intestinal lipid absorption and reduced hepatic lipid deposition and inhibited atherosclerotic lesion development in apoE^−/−^ mice fed a high-fat/high-cholesterol diet. These findings provide fundamental new insights into the biological effects of Nur77.

## Materials and Methods

### Materials

Cytosporone B (Csn-B) was purchased from the Sigma Chemical Company (St. Louis, MO, USA). The PrimeScript RT reagent Kit (Perfect Real Time) (DRR037A) (TaKaRa, Japan), SYBR® Premix Ex TaqTM II (Tli RNaseH Plus) (DRR820A) (TaKaRa, Japan) were obtained as indicated. All other chemicals were of the highest grade available from commercial sources.

### Animals and Diets

Male, 8-week-old apoE^−/−^ mice on a C57BL/6 background (purchased from the Laboratory Animal Center of Peking University, China) were randomized into eight groups: a baseline group (n = 10), control group (n = 10), vehicle group (n = 15), Csn-B group (n = 15), LV-Mock group (n = 15), LV-Nur77 group (n = 15), si-Mock group (n = 15) and si-Nur77 group (n = 15) and housed five per cage at 25°C on a 12-hour light/dark cycle. The baseline group was fed a standard chow diet, whereas the other mice were fed a high-fat, high-cholesterol diet containing 15% fat and 0.25% cholesterol (Laboratory Animal Center of Peking University, China). The vehicle group and the Csn-B group were treated daily with either vehicle (PEG400:Tween 80, 4∶1) or Nur77 agonist (Csn-B, 10 mg/kg body weight) by oral gavage (0.2 mL per mouse) for 12 weeks. The LV-Mock group and LV-Nur77 group were injected via the tail vein with control lentivirus (LV-Mock) or with lentivirus encoding mouse Nur77 (LV-Nur77). The si-Mock group and si-Nur77 group were injected via the tail vein with control lentivirus (si-Mock) or with lentivirus encoding mouse Nur77 (si-Nur77). Body weight was monitored at regular intervals. At week 12, the mice were sacrificed, blood was obtained, and tissues were collected for further analysis. Animal care and experimental procedures were approved by the Animal Experimental Committee at Nanfang Hospital.

### RNA Isolation and Real-time Quantitative PCR Analysis

Total RNA from mouse tissues or cultured cells was extracted using TRIzol reagent (Invitrogen) in accordance with the manufacturer’s instructions. Real-time quantitative PCR, using SYBR Green detection chemistry, was performed on the ABI 7500 Fast Real Time PCR system (Applied Biosystems, Foster City, CA, USA). Melt curve analyses of all real-time PCR products were performed and shown to produce a single DNA duplex. All samples were measured in triplicate and the mean value was considered for comparative analysis. Quantitative measurements were determined using the ΔΔCt method and GAPDH expression was used as the internal control. Primer sequences of genes for real-time PCR analyses are listed in Tables S1 and S2 in Materials and Methods S1.

### Statistical Analyses

Data are expressed as means ± standard deviations (SDs). Results were analyzed by one-way analysis of variance and the Student’s t-test using SPSS v13.0 statistical software (SPSS, Inc., Chicago, IL, USA). A probability (p) value <0.05 was considered statistically significant.

For other materials and methods, please refer to the Materials and Methods S1.

## Results

### 1. Nur77 Contributes to Lipid Loading, Lipid Content and Cholesterol Efflux

**Figure 1:**
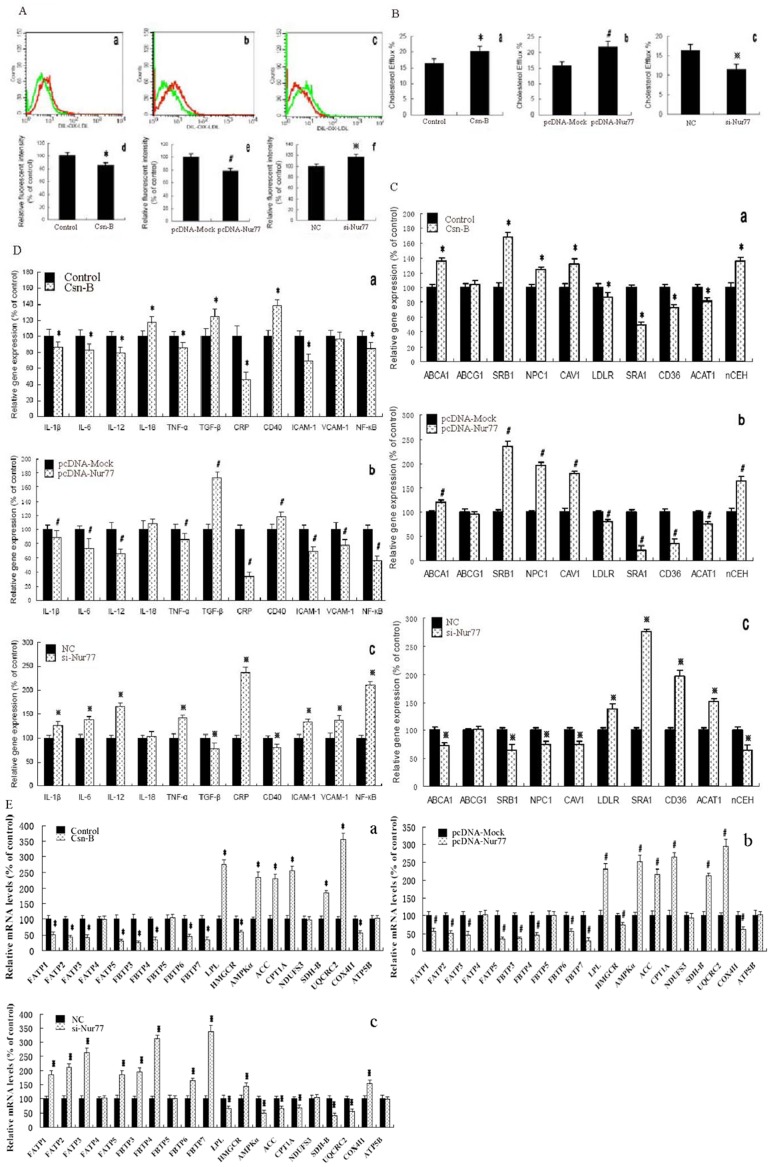
Effect of Nur77 on lipid loading, lipid content and cholesterol efflux. (**A**) a and d, THP-1 macrophages were treated with DMSO (Control) or 10 µg/ml Csn-B (Csn-B) for 24 h. b and e, THP-1 macrophages were treated with control (pcDNA-Mock) or recombinant plasmid over-expressing Nur77 (pcDNA-Nur77) for 48 h. c and f, THP-1 macrophages were treated with negative control (NC) or siRNAs against Nur77 (si-Nur77) for 48 h, and then incubated with 5 µg/ml DiI-labeled ox-LDL for 24 h, respectively. Uptake of DiI-labeled ox-LDL was analyzed by flow cytometry. a, Representative histogram of DiI-ox-LDL uptake in the presence of Csn-B (green peak) or DMSO (red peak). b, Representative histogram of DiI-ox-LDL uptake in the presence of pcDNA-Nur77 (green peak) or pcDNA-Mock (red peak). c, Representative histogram of DiI-ox-LDL uptake in the presence of si-Nur77 (green peak) or NC (red peak). d, e and f, DiI-ox-LDL uptake was significantly reduced in THP-1 macrophages treated with Csn-B and pcDNA-Nur77 while significantly increased in THP-1 macrophages treated with si-Nur77 as compared to controls, pcDNA-Mock and NC, respectively. (B, C, D and E) a, THP-1 macrophage-derived foam cells were treated with DMSO (Control) or 10 µg/ml Csn-B (Csn-B) for 24 h. b, THP-1 macrophage-derived foam cells were treated with control (pcDNA-Mock) or recombinant plasmids over-expressing Nur77 (pcDNA-Nur77) for 48 h. c, THP-1 macrophage-derived foam cells were treated with negative control (NC) or siRNAs against Nur77 (si-Nur77) for 48 h. (B) Cellular cholesterol efflux was analyzed by liquid scintillation counting assays as shown above. (C, D and E) Gene expression was measured by real-time quantitative PCR. The results are expressed as mean ± S.D. from three independent experiments, each performed in triplicate. * *p*<0.05 vs. control group, ^#^ *p*<0.05 vs. pcDNA-Mock group, and ^※^ <0.05 vs. NC group.

Previous work has shown Nur77 expression in human atherosclerotic lesion macrophages [10]. We first investigated the role of Nur77 during lipid loading in THP-1 macrophages, the effects of Nur77 on lipid content and cholesterol efflux in THP-1 macrophage-derived foam cells by treatment with Nur77 agonist Cytosporone B (Csn-B, 10 µg/ml), recombinant plasmid over-expressing Nur77 (pcDNA-Nur77) and siRNAs against Nur77 (si-Nur77). As shown (Fig. 1A), DiI-labeled ox-LDL uptake was obviously decreased by Csn-B (15.5%) and pcDNA-Nur77 (20.7%), while obviously increased by si-Nur77 (16.5%). Next, we examined the effects of Nur77 on cholesterol content and cholesterol efflux in THP-1 macrophage-derived foam cells by HPLC and liquid scintillation counting assays, respectively. As shown, cellular cholesterol content (Table 1) was decreased while cholesterol efflux (Fig. 1B) was increased when cells were treated with Csn-B and pcDNA-Nur77. On the contrary, cellular cholesterol content (Table 1) was increased while cholesterol efflux (Fig. 1B) was decreased when cells were treated with si-Nur77.

**Table 1:** Effect of Nur77 on cholesterol content in THP-1 macrophage-derived foam cells.

	Control	Csn-B	pcDNA-Mock	pcDNA-Nur77	NC	si-Nur77
TC (mg/dl)	498±36	385±33*	512±26	368±39 ^#^	505±33	712±41^ 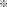 ^
FC (mg/dl)	196±21	157±17*	201±17	153±19 ^#^	198±21	270±26^ 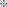 ^
CE (mg/dl)	302±27	228±23*	311±21	215±25 ^#^	307±27	442±31^ 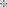 ^
CE/TC(%)	60.6	59.2	60.7	58.4	60.8	62.1

THP-1 macrophage-derived foam cells were divided into six groups and treated with DMSO (Control) or 10 µg/ml Csn-B (Csn-B) for 24 h, were treated with control (pcDNA-Mock) or recombinant plasmid over-expressing Nur77 (pcDNA-Nur77) for 48 h, and were treated with negative control (NC) or siRNAs against Nur77 (si-Nur77) for 48 h, respectively. Cellular cholesterol and cholesterol ester were extracted as described above. HPLC was performed to determine the cellular total cholesterol (TC), free cholesterol (FC) and cholesterol ester (CE). The results are expressed as mean ± S.D. from three independent experiments, each performed in triplicate. *p<0.05 vs. control group, ^#^ *p*<0.05 vs. pcDNA-Mock group, and ^
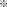
^ *p*<0.05 vs. NC group.

Subsequently, we aimed to understand the mechanism underlying the altered cellular lipid profile in response to the expression of Nur77 in THP-1 macrophage-derived foam cells by treatment with Csn-B, pcDNA-Nur77 and si-Nur77. For this purpose, we performed mRNA expression analysis of a panel of genes involved in lipid uptake, lipid transport and cholesterol efflux. As shown (Fig. 1C and E), Nur77 up-regulated genes included ATP-binding cassette A1 (ABCA1), scavenger receptor class B type 1 (SR-B1), Niemann–Pick C 1 protein (NPC1), Caveolin-1 (CAV-1), neutral cholesterol ester hydrolase (nCEH). Down-regulated genes through the expression of Nur77 included low density lipoprotein receptor (LDLR), 3-hydroxy-3-methylglutaryl-CoA reductase (HMGCR), cholesteryl ester transfer protein (CETP), scavenger receptor CD36 and SRA1. In addition, Nur77 had no effect on ATP-binding cassette G1 (ABCG1) mRNA expression and activity of HMGCR (data not shown). We also measured genes expression levels involving initial lipolysis of the triglyceride particles, fatty acid uptake and fatty acid metabolism. As shown (Fig. 1 E), Nur77 up-regulated genes included lipoprotein lipase (LPL), AMP- activated kinase α1 (AMPKα1), acetyl-CoA carboxylase (ACC), carnitine palmitoyltransferase 1A (CPT1A), succinate dehydrogenase iron-sulphur protein (SDH-B, Ip) and ubiquinol-cytochrome c reductase core protein 2 (UQCRC2). Down-regulated genes through the expression of Nur77 included fatty acid transport protein 1 (FATP1), FATP2, FATP3, FATP5, FABP3, FABP4, fatty acid binding protein 6 (FABP6), FABP7 and cytochrome c oxidase subunit IV isoform 1 (COX4I1). Moreover, Nur77 had no effect on FATP4, FABP5, NADH-ubiquinone oxidoreductase complex I Fe-S protein 3 (NDUFS3) and catalytic subunit of the mitochondrial H^+^-ATP synthase subunit β (ATP5B) mRNA expression. Furthermore, the gene expressions of FATP6, FABP1, FABP2, microsomal triglyceride transfer protein (MTP) and apolipoprotein B (apoB) were not detectable in THP-1 macrophage-derived foam cells through treatment with Csn-B, pcDNA-Nur77 and si-Nur77.

Since atherosclerosis is indeed a complex inflammatory disease and the macrophage foam cell is the major cell type involved in this progress [11], [12], we explored the effect of Nur77 on inflammatory gene expression in THP-1 macrophage-derived foam cells by treatment with Csn-B, pcDNA-Nur77 and si-Nur77. As shown in Fig. 1D, Nur77 up-regulated genes included transforming growth factor (TGF-β) and CD40, whereas down-regulated genes through the expression of Nur77 included interleukin-1β (IL-1β), interleukin-6 (IL-6), interleukin-12 (IL-12), tumor necrosis factor-α (TNF-α), C-reactive protein (CRP), intercellular adhesion molecule-1 (ICAM-1) and nuclear factor κB (NF-κB). In addition, Csn-B had no effect on vascular cell adhesion molecule (VCAM-1) mRNA expression, but up-regulated interleukin-18 (IL-18) mRNA expression. However, VCAM-1 mRNA expression was inhibited by pcDNA-Nur77, while enhanced by si-Nur77. Treatment with both pcDNA-Nur77 and si-Nur77 had no effect on IL-18 mRNA expression. Furthermore, the gene expressions of interferon-γ (INF-γ) and interleukin-10 (IL-10) were not detectable in THP-1 macrophage-derived foam cells through treatment with Csn-B, pcDNA-Nur77 and si-Nur77.

### 2. Nur77 Regulates Plasma Lipid Parameters and Circulating Cytokine Levels in apoE^−/−^ Mice

Because Nur77 is involved in regulation of cholesterol and lipid metabolism, we examined the terminal plasma lipid levels from experimental mice. As shown in Table 2, plasma high density lipoprotein cholesterol (HDL-C) showed a moderate 8.2% and 9.8% reduction in the Csn-B group and LV-Nur77 group as compared to their control groups, respectively. Concomitantly, plasma low density lipoprotein cholesterol (LDL-C) increased by 7.5% and 8.9% in the Csn-B group and LV-Nur77 group as compared to their control groups, respectively. In contrast, treatment with si-Nur77 led to a 10.9% increase in plasma HDL-cholesterol and an 8.5% reduction in plasma LDL-cholesterol as compared to the si-Mock group. However, no significant alternation occurred in apoA1, apoB, very low density lipoprotein cholesterol (VLDL-cholesterol), total triglyceride and cholesterol. Also, no difference occurred in body weights between groups at the end of the experiments.

**Table 2:** Effect of Nur77 on Serum Lipids and Lipoprotein Values in ApoE^−/−^ Mice.

	Baseline(n = 10)	Control(n = 10)	Vehicle(n = 10)	Csn-B(n = 10)	LV-Mock(n = 10)	LV-Nur77(n = 10)	si-Mock(n = 10)	si-Nur77(n = 10)
Body weight (g)	28.8±3.17	30.1±3.25	30.1±3.23	29.1±2.89	30.3±2.98	29.8±3.02	31.3±3.12	30.7±3.41
TG (mmol/L)	0.75±0.25	1.22±0.21 ^a^	1.22±0.25	1.22±0.34	1.22±0.16	1.21±0.43	1.22±0.36	1.23±0.49
TC (mmol/L)	16.98±3.25	27.78±3.10 ^a^	28.19±3.33	28.61±2.76	28.96±2.80	29.55±3.63	28.79±3.41	28.56±3.61
HDL-C (mmol/L)	7.25±1.12	10.33±1.06^ a^	10.45±1.12	9.59±1.11 ^b^	10.27±1.21	9.26±1.33^ c^	10.35±1.09	11.48±1.15^ d^
LDL-C (mmol/L)	9.19±1.28	16.58±1.67^ a^	16.89±1.73	18.15±1.67^ b^	17.83±1.52	19.42±2.17^ c^	17.58±2.06	16.20±1.96^ d^
VLDL-C (mmol/L)	0.54±0.17	0.87±0.11^ a^	0.85±0.32	0.87±0.23	0.86±0.31	0.87±0.41	0.86±0.26	0.88±0.15
ApoA-I (g/L)	0.04±0.02	0.05±0.01	0.04±0.01	0.04±0.02	0.05±0.02	0.04±0.02	0.04±0.01	0.04±0.02
ApoB (g/L)	0.13±0.02	0.15±0.02	0.15±0.03	0.14±0.02	0.16±0.03	0.15±0.01	0.15±0.02	0.16±0.03

Data are expressed as mean ±S.D. Unpaired *t*-test was used for comparisons between groups.

a, *p*<0.05 VS baseline; b, *p*<0.05 VS vehicle; c, *p*<0.05 VS LV-Mock; d, *p*<0.05 VS si-Mock.

**Figure 2:**
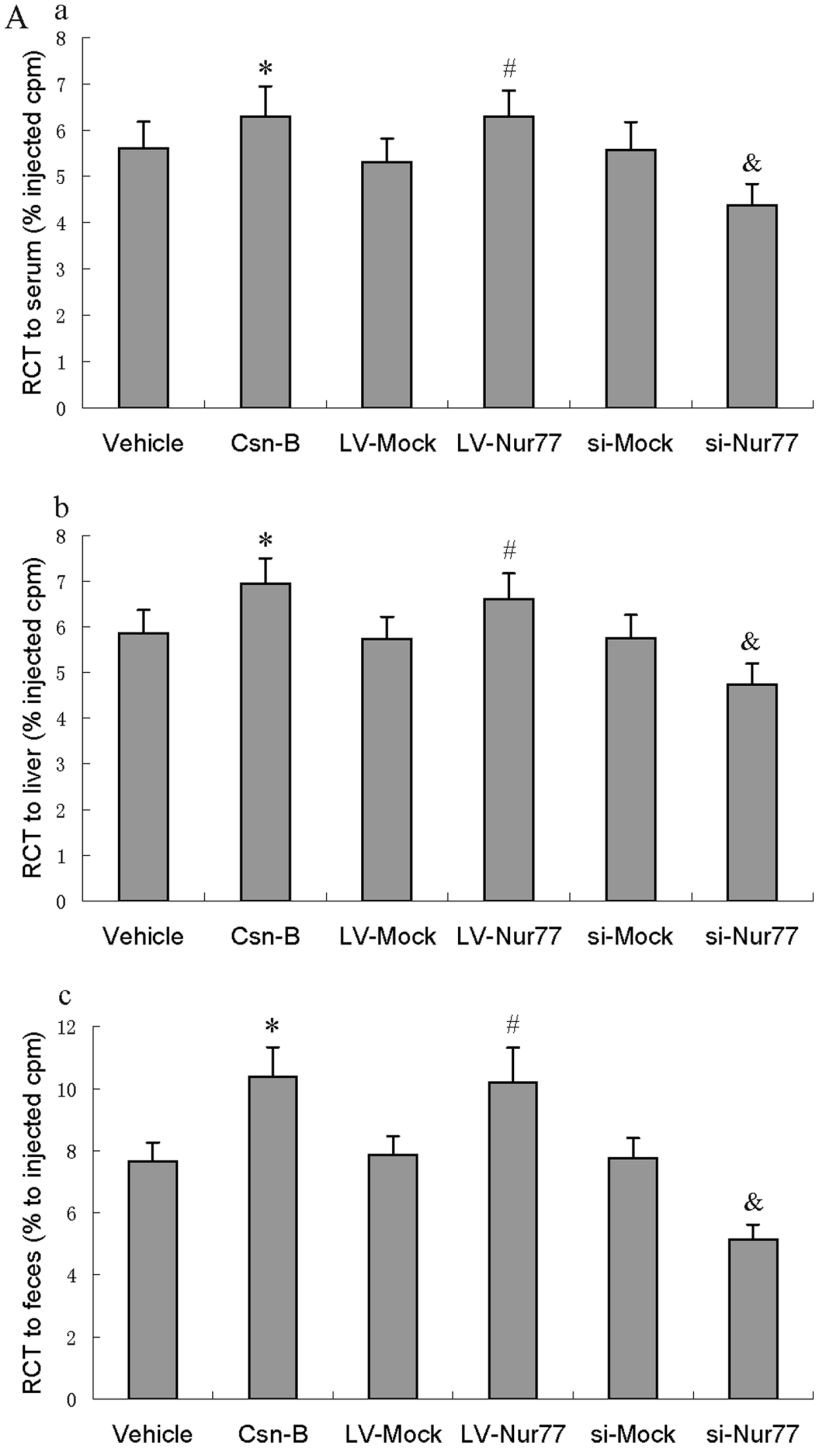
Effects of Nue77 on RCT. (**A**) After 12 weeks of the indicated treatment, apoE−/− mice were injected subcutaneously with 3H-cholesterol–labeled, Ac-LDL-loaded bone marrow-derived macrophages. Data are expressed as the percentage of the 3H-cholesterol tracer relative to that of total cpm tracer injected ± S.D. (a) plasma 3H-cholesterol tracer levels after 48 h. (b) Hepatic 3H-cholesterol tracer levels after 48 h. (c) Fecal 3H-cholesterol tracer levels. Feces were collected continuously from 0 to 48 h post-injection. All data are presented as the mean values ± S.D. n = 5. All experiments were performed in triplicate. * *p*<0.05 vs. vehicle group, ^#^ *p*<0.05 vs. LV-Mock group, and ^&^ *p*<0.05 vs. si-Mock group.

To investigate whether Nur77 treatment could regulate cholesterol transport from peripheral cells to the liver for further excretion into bile and feces, we performed an in vivo RCT assay to trace 3H-cholesterol from cholesterol-loaded macrophages ex vivo (Fig. 2A). Csn-B-, LV-Nur77- and si-Nur77-treated mice injected subcutaneously with cholesterol-loaded/3H-cholesterol–labeled bone marrow–derived macrophages showed 12.3% increase, 18.6% increase and 21.3% decrease in plasma 3H-cholesterol content at 48 h compared to that of control mice, respectively. Furthermore, Csn-B-, LV-Nur77- and si-Nur77-treated mice showed 18.5.3% increase, 15.2% increase and 17.6% decrease in the delivery of 3H-tracer to the liver and 35.5% increase, 29.6% increase and 33.9% decrease in 3H-sterols excreted into the feces, respectively. These results suggested that upregulated Nur77 expression could significantly enhanced the RCT pathway in vivo.

To investigate whether change in Nur77 expression could result in corresponding changes in plasma inflammatory cytokines, we conducted a series of ELISAs (Table 3). Consistent with the data of inflammatory gene expression in THP-1 macrophage-derived foam cells, treatment with both Csn-B and LV-Nur77 resulted in down-regulation of CRP concentrations by 41.5% and 48.7% in plasma, respectively. In agreement with these data, si-Nur77-mediated knockdown of Nur77 results in an up-regulation of CRP concentrations by 204.6% in plasma. However, no significant alteration occurred in levels of IL-1β, IL-6 and TNF-α between groups at the end of the experiments.

**Table 3:** Effect of Nur77 on Serum Cytokine Levels in ApoE^−/−^ Mice.

	Baseline (n = 10)	Control (n = 10)	Vehicle (n = 10)	Csn-B (n = 10)	LV-Mock (n = 10)	LV-Nur77 (n = 10)	si-Mock (n = 10)	si-Nur77 (n = 10)
IL-1β (pg/mL)	10.1±2.26	11.1±2.43	10.6±1.96	10.1±2.01	11.7±2.53	11.52±2.13	10.5±1.78	13.1±2.36
IL-6 (pg/mL)	55.2±4.36	60.37±5.46	62.49±4.45	61.33±3.78	62.37±4.43	60.37±5.85	59.45±4.56	62.27±5.79
TNF-α (pg/mL)	8.26±2.35	9.75±2.49	9.96±2.78	10.47±2.42	10.36±2.67	11.43±2.31	10.24±3.01	9.75±4.71
CRP (ng/mL)	25.29±5.83	30.31±6.76	31.27±5.98	18.29±5.15 ^a^	32.65±5.78	16.75±4.31^ b^	31.28±4.22	95.29±5.63 ^c^

Data are expressed as mean ±S.D. Unpaired *t*-test was used for comparisons between groups. a, *p*<0.05 vs vehicle; b, *p*<0.05 vs LV-Mock; c, *p*<0.05 vs si-Mock.

### 3. Nur77 Affects Hepatic Lipid Deposition in apoE^−/−^ Mice

**Figure 3:**
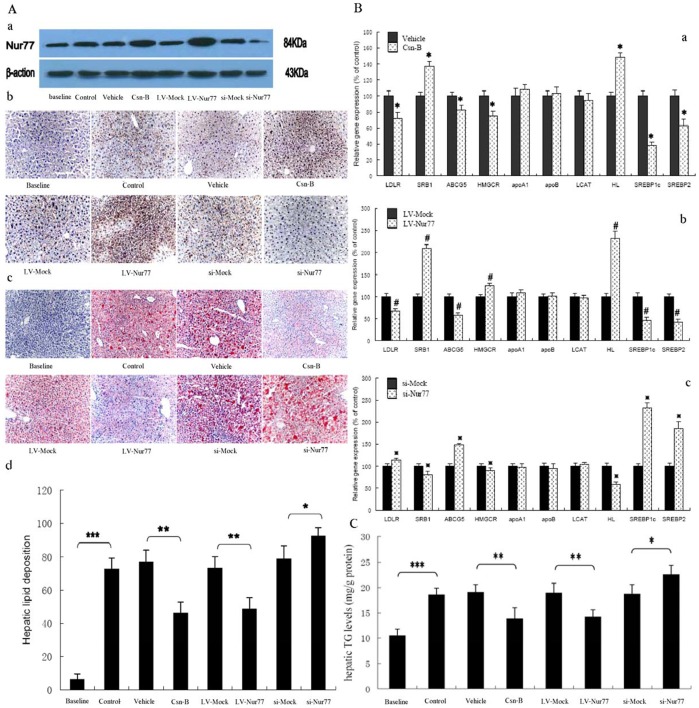
Effect of Nur77 on hepatic lipid deposition in apoE^−/−^ mice fed a high-fat/high cholesterol. (A, B and C) ApoE^−/−^ mice were randomized into eight groups–Baseline (fed a standard chow diet), Control (fed a high-fat/high diet), Vehicle (fed a high-fat/high diet and treated with PEG400: Tween 80, 4∶1), Csn-B (fed a high-fat/high diet and treated with Csn-B, 10 mg/kg body weight), LV-Mock (fed a high-fat/high diet and injected via the tail vein with control lentivirus), LV-Nur77 (fed a high-fat/high diet and injected via the tail vein with lentivirus encoding mouse Nur77), Si-Mock (fed a high-fat/high diet and injected via the tail vein with control lentivirus) and Si-Nur77 (fed a high-fat/high diet and injected via the tail vein with lentivirus encoding mouse Nur77). (A) a, Western blot analysis of the protein expression of Nur77 in mouse liver. b, Liver cryo-sections immunohistochemically stained. Original magnification: 200X. c, Liver cryo-sections were stained with Oil Red O and hemotoxylin. Original magnification: 100X. d, Hepatic lipid deposition was analyzed in apoE^−/−^ mice. (n = 5 per group). Values are the percent of the total lesion area stained and are expressed as mean ± S.D. * *p*<0.05; ** *p*<0.01; *** *p*<0.001. (B) a, b and c, gene expression was measured by real-time quantitative PCR. Data represent the mean values ± S.D. n = 5, performed in triplicate. * *p*<0.05 vs. vehicle group, ^#^ *p*<0.05 vs. LV-Mock group, and ^
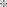
^ *p*<0.05 vs. si-Mock group. (C) Liver triglyceride levels were measured. Values are the mean ± S.D. n = 5, performed in triplicate. * *p*<0.05; ** *p*<0.01; *** *p*<0.001.

The protein expression of Nur77 in mouse liver was investigated by Western blot and immunohistochemistry analyses. As shown (Fig. 3A), minor expression levels were detectable in the baseline group, but expression of Nur77 was markedly increased in the control group. In addition, the Csn-B group and LV-Nur77 group had significantly higher expression of Nur77 than their control groups while the si-Nur77 group had a lower expression of Nur77 as compared to the si-Mock group. We next analyzed the effect of Nur77 on lipid content in the liver of apoE^−/−^ mice by Oil Red O staining. The lipid content in the liver was 39.9% and 33.6% less in mice treated with Csn-B and LV-Nur77, respectively, compared with mice of the control group (Fig. 3A). In contrast, the lipid content in the liver was 1.17-fold higher in mice treated with si-Nur77 in comparison to mice treated with si-Mock (Fig. 3A). Next, we investigated effects of Nur77 on liver ceramide levels and triglyceride (TG) contents in apoE^−/−^ mice and found that treatment with si-Nur77 resulted in an up-regulation of TG levels by 20.8% while treatment with both Csn-B and LV-Nur77 resulted in down-regulation of TG levels by 27.4% and 25.2%, respectively (Fig. 3C). Moreover, Nur77 had no effects on liver ceramide levels (data not shown).

To investigate the mechanisms of Nur77 reduction of hepatic lipid deposition, hepatic lipid metabolism gene expression levels in livers of apoE^−/−^ mice were analyzed by real time PCR (Fig. 3B). As shown, Csn-B and LV-Nur77 treatment progressively reduced gene levels of LDLR, ATP-binding cassette G5 (ABCG5), SREBP1c, SREBP2 and increased gene levels of SR-B1 and hepatic lipase (HL). In addition, treatment with si-Nur77 up-regulated gene expression of LDLR, ABCG5, SREBP1c and SREBP2 while SR-B1 and HL gene expressions were down-regulated. Although gene expression of HMG-CoA reductase (HMGCR) was repressed by treatment with Csn-B, the gene expression of HMGCR was increased by treatment with LV-Nur77 while decreased by treatment with si-Nur77. In addition, Nur77 had no effect on apoA1, apoB and LCAT expression. Next, we explored the effects of Nur77 on genes expression in HepG2 cells. Analogously, over-expression of Nur77 reduced gene expression of LDLR, ABCG5, HMGCR, SREBP1c and SREBP2. Moreover, siRNA for Nur77 repressed gene levels of SR-B1 and HL (data not shown). Furthermore, Nur77 had no effects on fatty acid synthase (FAS) mRNA expression both in HepG2 cells and in livers of apoE−/− mice, and also had no effects on apoB secretion in HepG2 cells (data not shown).

### 4. Nur77 Inhibits Gene Expression Involved in Intestinal Lipid Absorption

**Figure 4:**
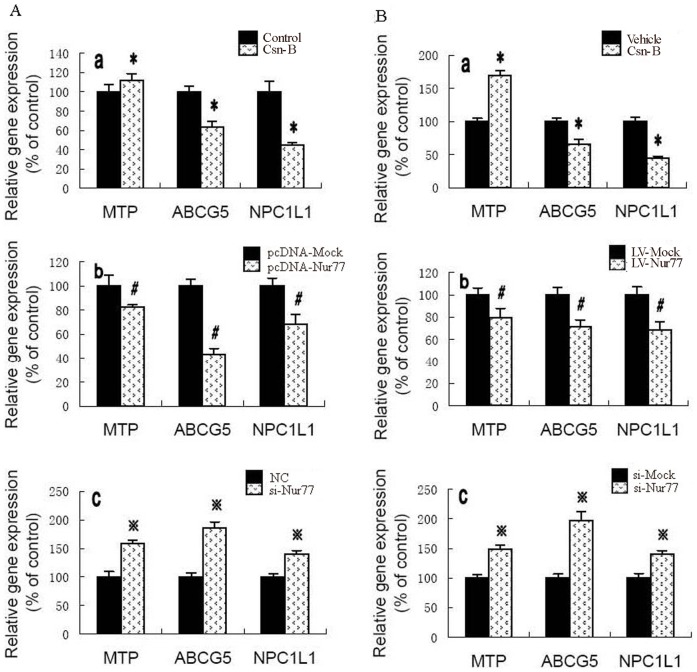
Effects of Nur77 on gene expression involved in intestinal lipid absorption. (A) a, Caco-2 cells were treated with DMSO (Control) or 10 µg/ml Csn-B (Csn-B) for 24 h. b, Caco-2 cells were treated with control (pcDNA-Mock) or recombinant plasmid over-expressing Nur77 (pcDNA-Nur77) for 48 h. c, Caco-2 cells were treated with negative control (NC) or siRNAs against Nur77 (si-Nur77) for 48 h. G expression was measured by real-time quantitative PCR. Results are expressed as mean ± S.D. from three independent experiments, each performed in triplicate. * *p*<0.05 vs. control group, ^#^ *p*<0.05 vs. pcDNA-Mock group, and ^
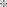
^ *p*<0.05 vs. NC group. (B) a, apoE^−/−^ mice were treated with PEG400:Tween 80, 4∶1(Vehicle) or with Csn-B, 10 mg/kg body weight (Csn-B). b, apoE^−/−^ mice were injected via the tail vein with control lentivirus (LV-Mock) or with lentivirus encoding human Nur77 (LV-Nur77). c, apoE^−/−^ mice were injected via the tail vein with control lentivirus (si-Mock) or with lentivirus encoding human Nur77 (si-Nur77). Gene expression was measured by real-time quantitative PCR. Data represent the mean values ± S.D. n = 5 performed in triplicate. * *p*<0.05 vs. vehicle group, ^#^ *p*<0.05 vs. LV-Mock group, and ^
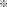
^ *p*<0.05 vs. si-Mock group.

The definitive identification of intestinal cholesterol transporters and elucidation of their role in the cholesterol absorption process will be fruitful to improve treatment strategies for the suppression of cholesterol absorption to reduce hypercholesterolemia and lower the risk of cardiovascular disease [13]. To address the possibility that expression levels of intestinal lipid transporters are affected by Nur77, we incubated Caco-2 cells with Csn-B, recombinant plasmid pcDNA-Nur77 and siRNAs against Nur77 (si-Nur77) (Fig. 4A). Csn-B and pcDNA-Nur77 treatment progressively reduced gene levels of MTP, Niemann-Pick C1-like 1 (NPC1L1) and ABCG5. On the contrary, si-Nur77 treatment markedly induced gene expression of MTP, NPC1L1 and ABCG5. To further investigate the Nur77 mechanisms affecting intestinal cholesterol absorption in apoE^−/−^ mice, gene expression in intestinal tissue of apoE^−/−^ mice were analyzed by real time PCR (Fig. 4B). We also found that over-expression of Nur77 reduced MTP, NPC1L1 and ABCG5 gene expression, while silenced Nur77 increased these gene levels.

### 5. Nur77 Reduces Plaque Formation in apoE^−/−^ Mice

**Figure 5:**
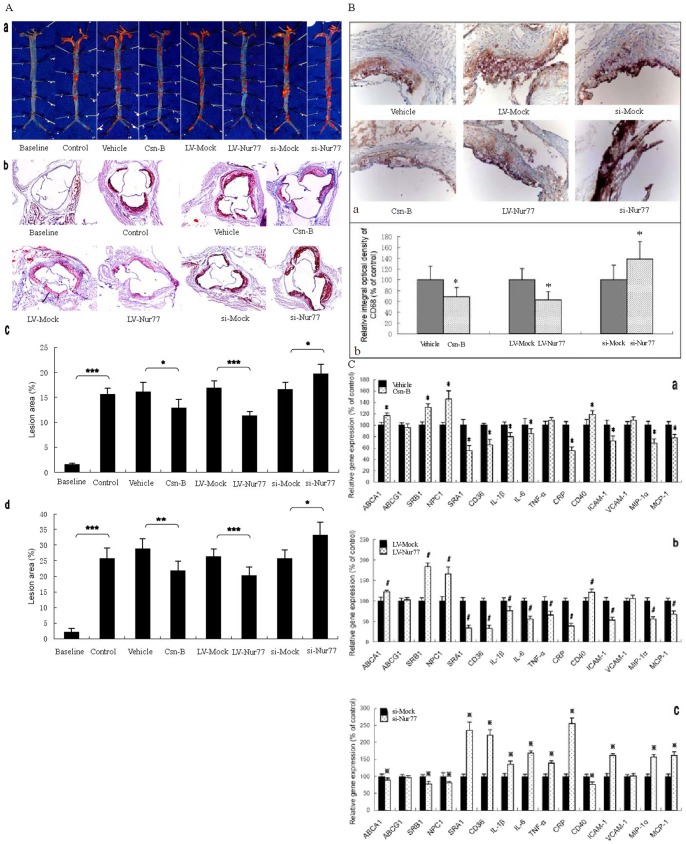
Effect of Nur77 on atherosclerosis initiation and development in apoE^−/−^ mice fed a high-fat/high cholesterol diet. (A, B and C) ApoE^−/−^ mice were randomized into eight groups–Baseline (fed a standard chow diet), Control (fed a high-fat/high diet), Vehicle (fed a high-fat/high diet and treated with PEG400:Tween 80, 4∶1), Csn-B (fed a high-fat/high diet and treated with Csn-B, 10 mg/kg body weight), LV-Mock (fed a high-fat/high diet and injected via the tail vein with control lentivirus), LV-Nur77 (fed a high-fat/high diet and injected via the tail vein with lentivirus encoding mouse Nur77), si-Mock (fed a high-fat/high diet and injected via the tail vein with control lentivirus) and si-Nur77 (fed a high-fat/high diet and injected via the tail vein with lentivirus encoding mouse Nur77). (A) a, Representative staining of aorta with Oil Red O. b, Representative staining of aortic valves with Oil Red O. c, Lesions of en face were analyzed in apoE^−/−^ mice (n = 5 per group). d, Lesions in aortic valves were analyzed in apoE^−/−^ mice (n = 5 per group). Data are mean ± S.D. * *p*<0.05; ** *p*<0.01; *** *p*<0.001. (B) (a) Cryo-sections of aortic valves from apoE−/− mice were immunohistochemically stained for the macrophage marker CD68. Original magnification: 200X (b) The integral optical density of CD68 in the aortic valves cryo-sections from apoE−/− mice was analyzed. * *p*<0.05 vs. control group, respectively. (C) Gene expression was measured by real-time quantitative PCR. Data are expressed as mean values ± SD; n = 5, performed in triplicate. * *p*<0.05 vs. vehicle group, ^#^ *p*<0.05 vs. LV-Mock group, and ^
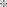
^ *p*<0.05 vs. si-Mock group.

To investigate the impact of Nur77 on atherogenesis in apoE^−/−^ mice, atherosclerotic lesions were evaluated by aortic valve section and en face analyses (Fig. 5A). Mice receiving Csn-B and LV-Nur77 showed a decrease in the average lesion area compared with their controls by both en face and aortic valve section analyses. On the contrary, si-Nur77-treated mice showed an increase in average lesion area compared with si-Mock-treated mice by both en face and aortic valve section analyses. Quantification of Oil Red O-stained aortic valve sections revealed that treatment with Csn-B and LV-Nur77 resulted in a significant decrease (24.4% and 23.2%, respectively) while treatment with si-Nur77 resulted in a significant increase (29.3%) in the lesion area in apoE^−/−^ mice when compared with their controls, respectively. To further document positive effects of Nur77 on atherosclerosis, Oil Red O-stained lesions in en face preparations of aortas were quantified. ApoE^−/−^ mice treated with Csn-B and LV-Nur77 led to a significant reduction (22.4% and 32.7%, respectively), while with treatment of si-Nur77 resulted in a significant increase (19.4%) in lesion areas as compared with their controls.

Subsequently, the composition of the observed plaques was determined by immunohistochemical staining techniques. The number of CD68^+^ cells (predominately macrophages and macrophage-foam cells) in the observed plaques of the Csn-B- and LV-Nur77-treated group was less than that in their control group respectively. Csn-B treatment (31.5%) and LV-Nur77 treatment (36.8%) resulted in a significant decrease in integral optical density of CD68^+^ cells in the plaque area (Fig. 5B). On the contrary, the number of CD68^+^ cells in the observed plaques of the si-Nur77-treated group was more than that in the control group. Si-Nur77 treatment resulted in a markedly increase (38.7%) in integral optical density of CD68^+^ cells in the plaque area (Fig. 5B).

To explore the mechanisms whereby Nur77 treatment inhibited plaque progression and stabilization, gene expression changes of the inflammatory molecules, adhesion molecules and molecules related to cholesterol metabolism were investigated in aortic tissues (Fig. 5C). In both Csn-B-treated and LV-Nur77-treated apoE^−/−^ mice, gene expression of SRA1, CD36, IL-1β, IL-6, CRP, ICAM-1, macrophage inflammatory protein-1α (MIP-1α) and monocyte chemo-attractant protein-1 (MCP-1) were markedly repressed, but gene expression of ABCA1, SR-B1, NPC1 and CD40 were up-regulated at week 12. On the contrary, we found that gene expression of SRA1, CD36, IL-1β, IL-6, CRP, ICAM-1, MIP-1α and MCP-1 were up-regulated, while gene expression of ABCA1, SRB1, NPC1 and CD40 were down-regulated in the aorta of si-Nur77-treated apoE^−/−^ mice. In addition, gene expression of TNF-α was reduced in LV-Nur77-treated apoE^−/−^ mice, while increased in si-Nur77-treated apoE^−/−^ mice. However, Csn-B-treated apoE-deficient mice had no change in TNF-α gene expression.

## Discussion

Multiple nuclear orphan receptors may participate in the atherosclerotic process, including Nur77, which is expressed in several tissues throughout the human body and recently has been found in human atherosclerotic lesions [10], [14]. Nur77 receptors have traditionally been considered to contribute to the atherosclerotic process via their ability to inhibit inflammatory cytokine secretion and foam cell formation [15]. However, there is limited data to support this hypothesis as it relates to Nur77. In the present study, anti-atherogenic effects of Nur77 were well documented. We observed that Nur77 could inhibit lipid loading in THP-1 macrophages, reduce lipid content and enhance cholesterol efflux in THP-1 macrophage-derived foam cells. We also found that Nur77 activation down-regulated expression of certain pro-inflammatory genes, but up-regulated others in THP-1 macrophage-derived foam cells and in the liver and aorta of apoE^−/−^ mice fed a high-fat/high cholesterol diet. In addition, we found that Nur77 could reduce intestinal cholesterol absorption gene expression in Caco-2 cells and in intestinal tissues of apoE^−/−^ mice fed a high-fat/high cholesterol diet, and also reduce lipid deposition in apoE^−/−^ mice fed a high-fat/high cholesterol diet. Moreover, we observed that Nur77 markedly promoted cholesterol transport from peripheral cells to the liver for further excretion into bile and feces, suppressed atherosclerotic plaque formation, despite slightly elevated plasma LDL-cholesterol and depressed plasma HDL-cholesterol in apoE^−/−^ mice fed a high-fat/high cholesterol diet. We presume that at least four mechanisms could account for the anti-atherogenic effects by Nur77, based on our data.

The first was accomplished through suppression of macrophage-derived foam cell Formation. The hallmark of atherosclerotic lesion development was the accumulation of macrophage foam cells [16]. The control of macrophage cholesterol homeostasis was of critical importance in the pathogenesis of atherosclerosis, as dysregulation of the balance between cholesterol influx, intracellular transport and cholesterol efflux will lead to excessive accumulation of cholesterol in macrophages and their transformation into foam cells [17], [18]. Our data suggested that Nur77 in THP-1 macrophage-derived foam cells contributes to anti-atherogenic effects through decreasing cellular cholesterol content because Nur77 was able to greatly inhibit expression of SRA1, CD36 and LDLR, which are important genes involved in modifying lipoprotein uptake [19]–[21], and to enhance expression of NPC1 and CAV1, which are crucial genes involved in mobilizing cholesterol from intracellular pools to the plasma membrane [21], [22], and to stimulate expression of ABCA1 and SRB1, which are key genes involved in mediating the transport of cholesterol across cellular membranes [21], [23], [24]. We also explored the changes in gene of molecules related to cholesterol metabolism in the aortas of apoE^−/−^ mice fed a high-fat/high cholesterol diet. We found that in both Csn-B-treated and LV-Nur77-treated apoE^−/−^ mice, gene expression of SRA1 and CD36 were markedly repressed, but gene expression of ABCA1, SRB1 and NPC1 were up-regulated. In addition, gene expression of SRA1 and CD36 were up-regulated, while gene expression of ABCA1, SRB1 and NPC1 were down-regulated in the aortas of si-Nur77-treated apoE^−/−^ mice. In addition, we performed an in vivo RCT assay that measures the integrated rate of movement of 3H-cholesterol from macrophages to the serum, liver, and feces. We found that Nur77 could increase the flux of cholesterol from cholesterol-loaded macrophages to all three of these compartments. Notably, this increase in RCT may reflect dual actions of Nur77. On the one hand, it may have ability to directly promote the efflux of cholesterol from macrophage foam cells by increasing expression of ABCA1 and SRB1 in these cells. On the other hand, it probably has ability to promote the uptake of HDL-C from the plasma to liver and direct it to biliary excretion by promoting SRB1 express in the liver. These results suggested that Nur77 contributes to an anti-atherogenic effect through reduced cholesterol uptake and enhanced cholesterol trafficking and cholesterol efflux, and thus reduced lipid content and suppressed foam cell formation.

The second was performed by repressing inflammatory gene expression. It is now well-accepted that atherosclerosis is not only merely a lipid disorder, but also a chronic inflammatory disease. Inflammatory processes are involved at all stages of the atherosclerotic process, from lesion initiation to plaque rupture [25], [26]. It has been reported that in cultured human THP-1 derived macrophages that over-expression of Nur77 could not only decrease oxidized LDL loading but also reduce inflammatory cytokine expression of IL-1β, IL-6 and IL-8 [10]. Here, we showed that Nur77 could up-regulate anti-atherogenic gene TGF-β and down-regulate pro-atherogenic genes including IL-1β, IL-6, IL-12, CRP, ICAM-1, VCAM-1 and NF-κB. To further explore the mechanisms whereby Nur77 treatment inhibited plaque progression and stabilization, changes in gene expression of inflammatory and adhesion molecules were investigated in the aorta in apoE^−/−^ mice fed a high-fat/high cholesterol diet. We observed that gene expression of IL-1β, IL-6, TNF-α, CRP, ICAM-1, MIP-1α and MCP-1 were markedly repressed, but gene expression of CD40 was up-regulated in the aortas of apoE^−/−^ mice through enhanced Nur77 expression. In addition, we showed that overexpression of Nur77 resulted in a markedly decrease in integral optical density of CD68^+^ cells in the plaque area. Our finding is in agreement with a previous work and data showing that cultured Nur77^−/−^ bone marrow–derived macrophages (BMM) exhibit a proinflammatory phenotype with enhanced expression of IL-12, IFN-γ, and SDF-1α and increased NO synthesis, suggesting that Nur77 has an anti-inflammatory function in these cells and thus suppresses the expression of inflammatory cytokines and chemokines [27]. These data provided support for the notion that modulating the expression of inflammatory molecules by Nur77 could block or retard the development of atherosclerotic lesions and thus positively influence disease outcomes.

The third was accomplished by decreasing hepatic lipid deposition. The liver is the major organ responsible for the production and degradation of apoB-100–containing lipoproteins [28], [29]. Based on the central role of the liver in determining plasma lipoprotein levels, several therapeutic strategies that act on hepatic lipid metabolism have been developed to reduce the susceptibility to atherosclerosis [30]. Initial experiments by Pols *et al*. demonstrated that adenoviral-mediated over-expression of Nur77 increased plasma LDL cholesterol and decreased HDL cholesterol while reducing hepatic triglyceride levels, which was thought to result from repression of the lipogenic transcription factor SREBP1c [31]. Subsequently, Chao et al. found that hepatic steatosis and increased SREBP1c expression occurred in Nur77-deficient mice fed a high-fat diet [32]. In this study, we further showed that over-expression of Nur77 could markedly reduce lipid content in the livers of apoE^−/−^ mice by Oil Red O-staining and decrease TG levels in the livers of apoE^−/−^ mice by enzymatic quantitative method. There are two metabolic pathways involved in the synthesis of TG including monoacylglycerol pathway and glycerol 3-phosphate pathway, which share the final step in converting diacylglycerol into TG [33]. Thus, Nur77 decreases liver TG content probably through reducing monoacylglycerol content and/or diacylglycerol content. However, this speculation and relative mechanism should be further investigated. We also found that Nur77 might inhibit gene expression of SREBP1c and SREBP2 both *in vivo* and *in vitro*. Consistent with these data, we speculated that enhancing the expression and/or activity of Nur77 in the liver may be beneficial in treating atherosclerosis with regard to its inhibitory effect on SREBP1c and SREBP2 activity and subsequent reduction in lipid accumulation in the liver.

The fourth was performed through inhibiting intestinal lipid absorption. Recent studies showed that ATP-binding cassette (ABC) transporters ABCG5 and ABCG8 may work together as an apical sterol export pump promoting active efflux of cholesterol and plant sterols from the enterocyte back into the intestinal lumen for excretion [34], [35]. Also, the newly identified Niemann-Pick C1-like 1 (NPC1L1) protein may play a critical role in the ezetimibe-sensitive cholesterol absorption pathway and might induce active influx of cholesterol from the intestinal lumen into the enterocyte [36], [37]. MTP, which is located within the lumen of the endoplasmic reticulum (ER) in absorptive enterocytes, plays a pivotal role in the assembly and secretion of triglyceride-rich, apolipoprotein B (apoB)-containing lipoproteins from intestine and also catalyzes the transport of triglycerides, cholesteryl esters, and phospholipids between membranes [38], [39]. Here, we showed that gene levels of MTP, NPC1L1 and ABCG5 can be down-regulated by Nur77 both *in vivo* and *in vitro*. These findings suggest that Nur77 has potential benefits in influencing dominant rate-limiting steps/factors in intestinal lipid absorption.

Additionally, other studies concerning the effect of Nur77 on vascular disease suggest that Nur77 may have a protective role in atherosclerosis through protection against smooth muscle cells (SMC)-rich lesion formation. SMCs under ’normal’ conditions are quiescent contractile cells that regulate blood flow, blood pressure, and vessel wall stability. Upon local inflammation or vascular damage, these functional SMCs become activated and start migrating to and proliferating in the intimal compartment of the vessel wall [40]. Arkenbout *et al* have shown that over-expression of Nur77 in both venous and arterial SMCs resulted in reduced proliferation while over-expression of a dominant-negative variant of Nur77 resulted in enhanced SMC proliferation. The Nur77-induced inhibition of SMC proliferation was accompanied with increased expression of the cell cycle inhibitor p27Kip1 and a decrease in the cell-cycle protein cyclin A [41]–[43]. Furthermore, transgenic mice over-expressing Nur77 in the arterial vessel wall under control of the arterial SMC-specific promoter-fragment of SM22a showed decreased vascular lesion formation, both after carotid artery ligation and upon femoral artery cuff placement [41], [43]. As a result, we presumed that Nur77 induced reduction of atherosclerosis progression in apoE^−/−^ mice fed a high-fat/high-cholesterol diet might also occur through a pathway where Nur77 might inhibit proliferation of SMCs and thus protected against SMC-rich lesion formation.

In summary, we have demonstrated that enhanced Nur77expression and activity in apoE^−/−^ mice decreased atherosclerotic plaque burden. This beneficial effect was accompanied by a reduction in macrophage- derived foam cells formation and gene expression of inflammatory and adhesion molecules in the aorta. Furthermore, Nur77 markedly promoted cholesterol transport from peripheral cells to the liver for further excretion into bile and feces, decreased hepatic lipid deposition and reduced intestinal lipid absorption. These findings established a new paradigm for the biological effects of Nur77 and might have major implications for treating atherosclerosis.

## Supporting Information

Materials and Methods S1 (DOC)
