# Supplementary material for: Nur77 Decreases Atherosclerosis Progression in apoE−/− Mice Fed a High-Fat/High-Cholesterol Diet
Source: PLoS One. 2014 Jan 31;9(1):e87313. doi: 10.1371/journal.pone.0087313 (PMC3909091; doi:10.1371/journal.pone.0087313)
Supplement: Materials and Methods S1 — (DOC) [file pone.0087313.s001.doc]

**Materials and Methods S1**

**Cell Culture**

Human monocytic THP-1 cells, HepG2 cells and Caco-2 cells were obtained from American Type Culture Collection (ATCC, Manassas, VA, USA). THP-1 cells were maintained in RPMI 1640 medium containing 10% fetal calf serum (FCS) in the presence of streptomycin (100μg/mL), penicillin (100 U/ml) and differentiated for 72 h with 100 nM phorbol 12-myristate 13-acetate (PMA). Macrophages were transformed into foam cells by incubation in the presence or absence of 50 μg/mL ox-LDL in serum-free RPMI1640 medium containing 0.3% bovine serum albumin (BSA) for 48 h. HepG2 cells and Caco-2 cells were grown in Dulbecco’s modified Eagle’s medium (DMEM) containing 10% FCS with streptomycin (100μg/mL) and penicillin (100 U/ml). All cells were incubated at 37°C, 5% CO_2_. Cells were seeded in 6- or 12-well plates or 60-mm dishes and grown to 80–90% confluence before use.

**Lentivirus Production and Tail Vein Injection**

Lentiviral constructs for mouse Nur77 were made as previously described ^1, 2^. Viral multiplicity of infection for liver infection was estimated based on *in vitro* primary hepatocyte transduction efficiency: 0.5 mL of undiluted viral stocks supplemented with Polybrene (7.5 μg/mL) was added to 10^5^ primary hepatocytes cultured in 12-well plates, and GFP-positive cells were counted 96 h after transduction. Anesthetized male, 8-week-old, apoE^-/-^ mice were injected with 150 μL of undiluted viral stocks supplemented with Polybrene (5 μg/mL) into the tail vein.

**Plasmid Construction and Transfection**

The PCR-XL-TOPO vector containing Nur77, vector PIRES2-EGFP, Platinum HIFI Taq polymerase and Accuprime Pfx DNA polymerase were purchased from Invitrogen Biotechnology (Shanghai, China). The pCDNA3.1(+) vector, competent DH5α cells, XhoI and EcoRI restriction enzymes and T4 DNA ligase were purchased from TaKaRa Biotechnology Co. Ltd. (Dalian, China). The fragment of EcoRI-Nur77-IRES-EGFP-XhoI was achieved from the PCR-XL-TOPO vector and PIRES2-EGFP vector by overlap extension PCR and was linked to pcDNA3.1 to create the recombinant plasmid pcDNA3.1-Nur77-IRES-EGFP. The inserted gene was identified by electrophoresis and sequencing. The recombinant plasmid was then transfected into the cultured cells by Lipofectamine 2000 (Invitrogen), and the over-expression effects of Nur77 was confirmed by RT-PCR and western blotting.

**Transfection with siRNA**

Short-interfering RNA (siRNA) specific for human Nur77 and nonsilencing control siRNA were synthesized by the Biology Engineering Corporation in Shanghai, China. Cells (2×10^6^/well) were transfected using Lipofectamine 2000. Forty-eight h post-transfection, real-time RT-PCR and Western blotting were performed. Based on the Western blot analysis, the Nur77 siRNA suppressed the expression of Nur77 proteins by 83%, 88% and 81% as compared to the control siRNA in THP-1 macrophage-derived foam cells, HepG2 cells and Caco-2 cells, respectively.

**Measurement of Serum Biochemical Parameters**

The serum concentrations of IL-1β, IL-6 and TNF-α were measured in duplicate using a commercial ELISA kit (R&D Systems, Minneapolis, MN, USA). Serum CRP amount was measured in duplicate using a commercial ELISA kit (Diagnostic System Laboratories, Webster, TX, USA). The serum apolipoprotein A1 (apoA1) and apoB100 concentrations were measured in duplicate using a commercial ELISA kit (Cusabio Biotech Co., Ltd., China). The T-Cho, TG, LDL-C, HDL-C and VLDL-C concentrations were determined enzymatically using an automated analyzer.

**Western Blot Analyses**

Cells were harvested and protein extracts prepared as previously described ^3^. Extracts were then subjected to Western blot analyses [10% SDS-polyacrylamide (SDS-PAGE); 30 *μ*g protein per lane] using rabbit anti-Nur77 (Santa Cruz Biotech, Santa Cruz, CA, USA) and goat β-actin (Santa, Cruz, Santa Cruz, CA, USA) antibodies. The proteins were visualized using a chemiluminescence method (ECL Plus Western Blotting Detection System; Amersham Biosciences, Foster City, CA, USA).

**Flow Cytometry**

PMA-differentiated THP-1 cells were treated with Nur77 agonist Cytosporone B (Csn-B, 10 μg/ml), recombinant plasmids over-expressing Nur77 (Ad-Nur77) and siRNAs against Nur77 (si-Nur77) as indicated, then fluorescent-tagged Dil-oxLDL was added, and the cells were incubated for 24 h. Adherent cells were harvested, washed three times with phosphate buffer saline (PBS). Analysis was performed on a fluorescent activated cell sorting (FACS) calibur flow cytometer (Becton Dickinson, Franklin Lakes, NJ, USA) with Cell Quest Pro software (BD Biosciences, San Jose, CA, USA).

**High Performance Liquid Chromatography Assays**

High performance liquid chromatography (HPLC) analysis was conducted as previously described ^4^. The sterol analyses were performed using a HPLC system (model 2790, controlled with Empower Pro software; Waters Corp., Milford, MA, USA). Absorbance at 216 nm was monitored. Data were analyzed with TotalChrom software from PerkinElmer (Waltham, MA, USA).

**Cellular Cholesterol Efflux Experiments**

Cells were cultured and treated with Nur77 agonist Cytosporone B (Csn-B, 10 μg/ml), recombinant plasmids over-expressing Nur77 (pcDNA-Nur77) and siRNAs against Nur77 (si-Nur77), as indicated above. Then, they were labeled with 0.2 μCi/ml [^3^H]cholesterol. After 72 h, cells were washed with PBS and incubated overnight in RPMI 1640 medium containing 0.1% (w/v) BSA to allow equilibration of [^3^H]cholesterol in all cellular pools. Equilibrated [^3^H]cholesterol-labeled cells were washed with PBS and incubated in 2 ml of efflux medium containing RPMI 1640 medium and 0.1% BSA with 25 μg/ml human plasma apoA-I. A 150 μl sample of efflux medium was obtained at the times designated and passed through a 0.45-μm filter to remove any floating cells. Monolayers were washed twice with PBS, and cellular lipids were extracted with isopropanol. Medium and cell-associated [^3^H]cholesterol was then measured by liquid scintillation counting. Percent efflux was calculated by the following equation: [total media counts/(total cellular counts + total media counts)]×100%.

**Liver Triglyceride Measurement**

Liver tissues (100 mg) were homogenized in 1 ml of PBS, then 200 μl of liver homogenate was mixed with 400 μl of PBS and 850 μl of chloroform/methanol (2:1, v/v) solution, vortexed, and spun at 10,000 rpm for 10 min. Lower organic layer was dried under nitrogen gas. Triglycerides were assayed by enzymatic methods (Wako).

**In Vivo RCT Assay**

Bone marrow–derived macrophages were prepared from C57BL/6 mice as previously described^5^. Bone marrow was isolated and cells were plated overnight in DMEM supplemented with 10% FBS and 15% L-929 conditioned media. Non-adherent cells were removed and cultured for an additional 6 days to allow for macrophage differentiation. For the RCT assays, bone marrow-derived macrophages (BMDMs) were washed twice and incubated with 37.5 μg/mL of acetylated LDL (Ac-LDL) and 5 μCi/mL of 3H-cholesterol for 24 h as described previously^6^. Cells were resuspended in ice-cold DMEM and 3×10^6^ cells were injected subcutaneously into individually housed mice treated with vehicle, Csn-B, LV-Mock, LV-Nur77, si-Mock and si-Nur77 for 12 weeks as described above. Prior to injection, an aliquot of cells was counted using liquid scintillation counting to measure baseline radioactivity. Blood was obtained by cardiac puncture after 48 h at sacrifice. An aliquot of plasma was used for liquid scintillation counting immediately. Feces were collected for 48 h after injection and homogenized in 50% NaOH overnight, after which an aliquot was used for liquid scintillation counting. At sacrifice, liver samples were collected and incubated with hexane/isopropanol (3:2) for 48 h and then dried overnight. Lipids were resolubilized in liquid scintillation fluid, and radioactivity was counted. RCT to plasma, liver, and feces was calculated as a percentage of total radioactivity injected at baseline.

**En Face Plaque Area**

For en face analysis, aortas from different groups were opened longitudinally from the heart to the iliac arteries, and lesions were stained with Oil Red O. En face aortic lesion areas were digitized by a Nikon S6 digital camera, analyzed using Image-Pro Plus image analysis software (Media Cybernetics, Bethesda, MD, USA), and expressed as the percentage of the total aortic surface area covered by lesions.

**Atherosclerotic Lesion Assessment of Aortic Sinus**

The upper portion of the heart and proximal aorta were obtained, embedded in Optimal Cutting Temperature (OCT) compound (Fisher, Tustin, CA), and stored at -70^o^C. Serial 10-μm thick cryosections of aorta, beginning at the aortic root, were collected over a distance of 400 μm. Sections were stained with Oil Red O. The Oil Red O-positive areas in digitized color images of stained aortic root sections (three equally spaced sections per mouse; n = 5 per group) were quantified using Image-Pro Plus image analysis software (Media Cybernetics), and the data are expressed as percent of total section area.

**Hepatic Lipid Deposition Assessment by Oil Red O Staining**

Hepatic lipid deposition was assessed in samples embedded in OCT compound by Oil Red O staining. Briefly, liver cryosections were fixed for 10 min in 60% isopropanol and stained with 0.3% Oil Red O in 60% isopropanol for 30 min and subsequently washed with 60% isopropanol. Sections were counterstained with Gill’s hematoxylin, washed with acetic acid solution (4%), and mounted with aqueous solution. Once stained, sections were quantified by histomorphometry.

**Immunohistochemistry**

For this procedure, each frozen liver tissue or aortic root sample was sectioned to 5-μM thicknesses and fixed to microscope slides. Sections of frozen tissue specimens were mounted on polyl-lysine (Sigma)-coated slides, air dried, and fixed with acetone. Immunohistochemical staining was performed for Nur77 and CD68 using rabbit polyclonal antibody to Nur77 and CD68 at a dilution of 1:100 (Abcam, Cambridge, MA, USA). Images were acquired and quantitated on an Olympus BX50 microscope using Optimis software (Version 6.2) and digitized using a color video camera (three-charge coupled device; JVC, Wayne, NJ, USA).

**Table S1.** Primer sequences for human mRNAs measured by real-time PCR (The primer sequences are listed from 5’ to 3’).

| mRNA | Forward Primer | Reverse Primer |
| --- | --- | --- |
| ABCA1 | GTCCTCTTTCCCGCATTATCTGG | AGTTCCTGGAAGGTCTTGTTCAC |
| ABCG1 | TCTTCGTCAGCTTCGACACCA | TCTCGTCGATGTCACAGTGCAG |
| SR-B1 | ATGAAATCTGTCGCAGGCATTG | TGCATCACCTTGGGCATCA |
| NPC1 | AGCCACATAACCAGAGCGTTCAC | CCATGGCCAAATACATCCTGAAG |
| CAV-1 | CCTCAACGATGACGTGGTCAA | TCGTCACAGTGAAGGTGGTGAAG |
| SRA1 | TTTGGAACAGGCATTGGAAG | GCGGTGGATGTCATCTGCT |
| CD36 | GAGAACTGTTATGGGGCTAT | TTCAACTGGAGAGGCAAAGG |
| LDLR | GGCAGTGTGACCGGGAATATG | TTCGCCGCTGTGACACTTG |
| ABCG5 | CCTTGACAGGCACTCAAATG | TTTCTCAATGAATTGAATTCCTT |
| NPC1L1 | GGGTGGATGACTTCATTGACTGG | CATCGTGATGCTCATGCAGTTC |
| MTP | GCAGATGGACAAGGATGAAGCTC | GCGGGAATTCACATCCTGCTA |
| apoA1 | ACTGTGTACGTGGATGTGCTCAAAG | CACGCTGTCCCAGTTGTCAAG |
| nCEH | TCTGGAAGTTTCAACAGCCCAAG | AGGCAGAGATCCTCAACTCAAATCA |
| ACAT1 | TAACAGCTGCCAATGCCAGTACA | GGTTCTACAGCAGCGTCAGCAA |
| CD40 | CACTGTACGAGTGAGGCCTGTGA | TTGCACAACCAGGTCTTTGGTC |
| ICAM-1 | TGTATGAACTGAGCAATGTGCAAGA | CACCTGGCAGCGTAGGGTAA |
| VCAM-1 | CGTGATCCTTGGAGCCTCAAATA | GACGGAGTCACCAATCTGAGCA |
| CRP | AATGTGAACATGTGGGACTTTGTG | CGCCAGTTCAGGACATTAGGAC |
| TNF-α | CACTCCAGCAGCTCAAGCAGA | GTGCACCAGCTCAATGGTTTC |
| INF-γ | CTTTAAAGATGACCAGAGCATCCAA | GGCGACAGTTCAGCCATCAC |
| TGF-β1 | GCGACTCGCCAGAGTGGTTA | GTTGATGTCCACTTGCAGTGTGTTA |
| IL-1β | CCAGGGACAGGATATGGAGCA | TTCAACACGCAGGACAGGTACAG |
| IL-6 | AAGCCAGAGCTGTGCAGATGAGTA | TGTCCTGCAGCCACTGGTTC |
| IL-10 | GAGATGCCTTCAGCAGAGTGAAGA | AGTTCACATGCGCCTTGATGTC |
| IL-12β | TGTCACCAGCAGTTGGTCATCTC | CTCACTGCTCTGGTCCAAGGTC |
| IL-18 | CTGCCACCTGCTGCAGTCTA | TCTACTGGTTCAGCAGCCATCTTTA |
| NF-κ b | GCCTCCACAAGGCAGCAAATA | CACCACTGGTCAGAGACTCGGTAA |
| apoB | TTCTCAAGAGTTACAGCAGATCCA | TGGAAGTCCTTAAGAGCAACTAACA |
| FABP1 | CAGTGGTTCAGTTGGAAGGTGA | TCTTGAAGACAATGTCACCCAATGT |
| FABP6 | CAACAAGTTCACTGTTGGCAAGG | CACGATCTCTGAGGTCTGGTGATAG |
| FABP7 | AGACAAAGTGGTCATCAGGACTCTC | TGTGAACAAGTTTGTCTCCATCCAG |
| ATP5B | GGTCCTGAGACTTTGGGCAGAA | CCTCAGCATGAATGGGAGCA |
| COX4I1 | GCCCATGTCAAGCACCTGTC | CCCTGTTCATCTCAGCAAAGCTC |
| SDH-B | GGAGGCAACACTCTAGCTTGCAC | CAGATACTGCTGCTTGCCTTCCT |
| UQCRC2 | TCAAGATAACCCGTGGAATTGAAG | GCTGAGGCTGAAGGTCAGCTA |
| FATP2 | TCGGTATTTATGCAACTCACCACA | GCCTTCAGTGGCAGCATAGAAC |
| FABP3 | CCACACTTGTGCGGGAGCTA | GAAATGAGGCAATGTGGTGCTG |
| NDUFS3 | CTGGTTGACTTGACAGCAGTGGA | AGCGCAGAGACAACAGGTTGTAGA |
| FATP4 | ACTGGCAACTGTGACCTGGAG | CTTTCACAATAGCCGGGTCAA |
| FATP5 | ACGGTCCTGCCTCTGTACCA | TCGCCCACATACAGGATCAC |
| FATP6 | TGGCCTTTCTCAACACCAACAT | TTTCTGAGAGGCTTGGAAGGAT |
| FATP3 | ATGGGCCAGTGCCAGGATAC | GTGGACACCACACAGCTGATAGAAG |
| FABP2 | AAAGCTGAATACTGCGTGTTCTCA | CCAACCCTCCAACTCTTCCA |
| CPT1A | CGCTCATGGTGAACAGCAACTA | TGTAAAGCAGGATGGCATGGA |
| FABP4 | TCCACGAGAGTTTATGAGAG | CCCACAGAATGTTGTAGAGT |
| FABP5 | AACACAGTTTTCTTGTACCC | CAATTTTCTTGTTATTGTGC |
| FATP1 | TCTGAAGCTGTTCCTCTACT | AGGTTCCAGTTTCTCAGTC |
| ACC | ACTACAGGTTCTTTGTTCGT | AGGAAGATGTGGTTACAGTC |
| AMPKα1 | GACTGCTACTCCACAGAGATCG | TCAGCATCTAGGTCACTCCTTT |

**Table S2.** Primer sequences for mouse mRNAs measured by real-time PCR (The primer sequences are listed from 5’ to 3’).

| mRNA | Forward Primer | Reverse Primer |
| --- | --- | --- |
| ABCA1 | TGAAGCCTGTCCAGGAGTTC | ATGACAAGGAGGATGGAAGC |
| ABCG1 | CAAGACCCTTTTTGAAAGGGATCTC | GCCAGAATATTCATGAGTGTGGAC |
| SR-B1 | TGCAGCTGAGCCTCTACATCAA | AACCACAGCAACGGCAGAACTA |
| NPC1 | TGAATGCGGTCTCCTTGGTC | CTCACTCGGCTTCCTTTGGTA |
| SRA1 | AAGAACAAGCGCACGTGGAA | ACCAGTTTGTCCAGTAAGC |
| CD36 | TTTCCTCTGACATTTGCAGGTTCTA | AAAGGCATTGGCTGGAAGGAA |
| LDLR | AATGAGGAGCAGCCACATGGTA | TGTTGATGTTCTTCAGCCGCCA |
| ABCG5 | TGGCCCTGCTCAGCATCT | ATTTTTAAAGGAATGGGCATCTCTT |
| NPC1L1 | GAGAGCCAAAGATGCTACTATCTTCA | CCCGGGAAGTTGGTCATG |
| MTP | GTGGAGGAATCCTGATGGTGA | TGATCTTAGGTGTACTTTTGCCC |
| apoA1 | GGCAGAGACTATGTGTCCCAGTT | CCCAGTTTTCCAGGAGATTCAG |
| CETP | ATGGTGGGAATGGGTCAGAAG | CACGCAGCTCATTGTAGAAGG |
| LCAT | TATGTGATGGGGCTGCCTG | GCTGTGGTTGTAGACAATCCTG |
| HL | TATGGCTGGAGGAATCTG | GCATCATCAGGAGAAAGG |
| MCP-1 | CAGCCAGATGCAGTTAACG | TCTCTCTTGAGCTTGGTGAC |
| MIP-1α | ACCTGGAACTGAATGCCTGAGA | GCTTATAGGAGATGGAGCTATGCA |
| CD40 | AGCAGGGACTTTGGAGTGACTTG | TGGCCATCGTGGAGGTACTG |
| ICAM-1 | AACTGTGGCACCGTGCAGTC | AGGGTGAGGTCCTTGCCTACTTG |
| VCAM-1 | GCCACCCTCACCTTAATTGCTATG | TGTGCAGCCACCTGAGATCC |
| CRP | TTGATGCAAAGCAGTCTTTGGTG | AGTTCAGTGCCCGCCAGTTC |
| TNF-α | TCTTCTGTCTACTGAACTTCG | GAAGATGATCTGAGTGTGAGG |
| IL-1β | CAACCAACAAGTGATATTCTCCATG | GATCCACACTCTCCAGCTGCA |
| IL-6 | CTGCAAGAGACTTCCATCCAGTT | GAAGTAGGGAAGGCCGTGG |
| SREBP2 | GTGGAGCAGTCTCAACGTCA | TGGTAGGTCTCACCCAGGAG |
| SREBP-1c | GTTACTCGAGCCTGCCTTCAGG | CAAGCTTTGGACCTGGGTGTG |
| FAS | GTATGTGACCACTGTGAGAA | CCACTAAACTGAGCCTACTT |
| apoB | AGCATGTGTCTGAAGCTGTCTGTG | GAAGCGACTGTTGATCTTAGGTGTG |

**References:**

**1.** Fumoto T, Yamaguchi T, Hirose F, Osumi T (2007)Orphan nuclear receptor Nur77 accelerates the initial phase of adipocyte differentiation in 3T3-L1 cells by promoting mitotic clonal expansion. J Biochem 141:181-192.

**2.** Seppen J, Rijnberg M, Cooreman MP, Oude ER (2002) Lentiviral vectors for efficient transduction of isolated primary quiescent hepatocytes. J Hepatol 36:459-465.

**3.** Cho SD, Yoon K, Chintharlapalli S, Abdelrahim M, Lei P, et al. (2007) Nur77 agonists induce proapoptotic genes and responses in colon cancer cells through nuclear receptor-dependent and nuclear receptor-independent pathways. Cancer Res 67:674-683.

**4.** Hu YW, Ma X, Li XX, Liu XH, Xiao J, et al. (2009) Eicosapentaenoic acid reduces ABCA1 serine phosphorylation and impairs ABCA1-dependent cholesterol efflux through cyclic AMP/protein kinase A signaling pathway in THP-1 macrophage-derived foam cells. Atherosclerosis 204:e35-e43.

5. Zhang Y, Da Silva JR, Reilly M, Billheimer JT, Rothblat GH, et al. (2005) Hepatic expression of scavenger receptor class B type I (SR-BI) is a positive regulator of macrophage reverse cholesterol transport in vivo. J Clin Invest 115:2870-2874.

6. Inoue S, Egashira K, Ni W, Kitamoto S, Usui M, et al. (2002) Anti-monocyte chemoattractant protein-1 gene therapy limits progression and destabilization of established atherosclerosis in apolipoprotein E-knockout mice. Circulation 106:2700-2706.
